# Association of low ficolin-2 concentration in cord serum with respiratory distress syndrome in preterm newborns

**DOI:** 10.3389/fimmu.2023.1107063

**Published:** 2023-01-17

**Authors:** Gabriela Gajek, Anna S. Świerzko, Dariusz Jarych, Damian Mikulski, Paulina Kobiela, Karolina Chojnacka, Maja Kufelnicka-Babout, Agnieszka Szala-Poździej, Jędrzej Chrzanowski, Katarzyna Sobczuk, Wojciech Fendler, Misao Matsushita, Iwona Domżalska-Popadiuk, Jan Mazela, Jarosław Kalinka, Hideharu Sekine, Maciej Cedzyński

**Affiliations:** ^1^ Laboratory of Immunobiology of Infections, Institute of Medical Biology, Polish Academy of Sciences, Łódź, Poland; ^2^ Department of Biostatistics and Translational Medicine, Medical University of Łódź, Łódź, Poland; ^3^ Department of Neonatology, Medical University of Gdańsk, Gdańsk, Poland; ^4^ II Department of Neonatology, Poznań University of Medical Sciences, Poznań, Poland; ^5^ Department of Perinatology, First Chair of Gynecology and Obstetrics, Medical University of Łódź, Łódź, Poland; ^6^ Department of Applied Biochemistry, Tokai University, Hiratsuka, Kanagawa, Japan; ^7^ Department of Neonatology, Poznań University of Medical Sciences, Poznań, Poland; ^8^ Department of Immunology, Fukushima Medical University, Fukushima, Japan

**Keywords:** *FCN2*, ficolin-2, newborn, prematurity, preterm, respiratory distress syndrome (RDS)

## Abstract

**Introduction:**

Ficolin-2 is a serum pattern recognition molecule, involved in complement activation *via* the lectin pathway. This study aimed to investigate the association of ficolin-2 concentration in cord blood serum with complications related to premature birth.

**Methods:**

546 premature neonates were included. The concentration of ficolin-2 in cord blood serum was determined by a sandwich TRIFMA method. *FCN2* genetic variants were analysed with RFLP-PCR, allele-specific PCR, Sanger sequencing or allelic discrimination using TaqMan probes method.

**Findings:**

Cord blood serum ficolin-2 concentration correlated positively with Apgar score and inversely with the length of hospitalisation and stay at Neonatal Intensive Care Unit (NICU). Multivariate logistic regression analysis indicated that low ficolin-2 increased the possibility of respiratory distress syndrome (RDS) diagnosis [OR=2.05, 95% CI (1.24-3.37), p=0.005]. Median ficolin-2 concentration was significantly lower in neonates with RDS than in premature babies without this complication, irrespective of *FCN2* gene polymorphisms localised to promoter and 3’untranslated regions: for patients born <33 GA: 1471 ng/ml *vs.* 2115 ng/ml (p=0.0003), and for patients born ≥33 GA 1610 ng/ml *vs*. 2081 ng/ml (p=0.012). Ficolin-2 level was also significantly lower in neonates requiring intubation in the delivery room (1461 ng/ml *vs.* 1938 ng/ml, p=0.023) and inversely correlated weakly with the duration of respiratory support (R=-0.154, p<0.001). Interestingly, in the neonates born at GA <33, ficolin-2 concentration permitted differentiation of those with/without RDS [AUC=0.712, 95% CI (0.612-0.817), p<0.001] and effective separation of babies with mild RDS from those with moderate/severe form of the disease [AUC=0.807, 95% CI (0.644-0.97), p=0.0002].

**Conclusion:**

Low cord serum ficolin-2 concentration (especially in neonates born at GA <33 weeks) is associated with a higher risk of developing moderate/severe RDS, requiring respiratory support and intensive care.

## 1 Introduction

Preterm birth is often associated with multiple pregnancies, infections and gestational diabetes, however most (even >60%) cases are unexplained (1). Common prematurity-associated complications include neonatal sepsis, necrotizing enterocolitis (NEC), intraventricular haemorrhage, patent ductus arteriosus (PDA) and retinopathy. Due to smaller surface area for gas exchange, thicker blood-gas barrier and fewer type II alveolar epithelial cells, respiratory problems are more common in preterm than in term infants (2) and are the main reasons for morbidity and mortality in premature babies. If not recognised and managed quickly, respiratory disease can escalate to respiratory failure and cardiopulmonary arrest (3). The similarities in clinical signs for common causes of respiratory distress like pneumonia, transient tachypnea of neonates (TTN), respiratory distress syndrome (RDS) and meconium aspiration syndrome (MAS) make it difficult to distinguish between them (4).

Human lung development begins between the 4^th^ and 7^th^ weeks of pregnancy and the formation of alveoli continues until early postnatal life (5). RDS is a multifactorial and complex disease associated with inadequate pulmonary surfactant production by immature lungs, resulting in microatelectasis, severe hypoxia, and acidosis (3, 6). The development of RDS may be caused by inadequate clearance of fetal lung liquid as well (7). Genetic susceptibility to RDS in preterm infants has also been demonstrated (8). RDS long-term complications include bronchopulmonary dysplasia (BPD) and asthma. In term and post-mature neonates, respiratory distress may be associated with meconium aspiration syndrome (MAS), and with a higher incidence of asthma in later life.

The complement system is a crucial mediator of the immune response to infection. It interacts with other innate mechanisms as well as acquired immunity, and engages in cross-talk with other endogenous cascades, like the coagulation network (9). It also contributes to cell homeostasis (10), tissue development and repair (11, 12), and reproduction (13). Low activity of classical, lectin or alternative routes of complement in sera of premature babies may enhance their vulnerability to infections due to impaired clearance of pathogens *via* opsonophagocytosis or direct lysis of pathogens. The lectin pathway (LP) of complement activated by microbial glycoconjugates may play a crucial role in the protection of neonates from infections due to their poor response to T-independent polysaccharide antigens and limited transplacental transfer of maternal antibodies [reviewed in (14)].

Knowledge concerning associations of LP with complications related to prematurity is still limited. Several reports have documented correlations of LP-specific factors in serum with gestational age [reviewed in (14)]. One of the pattern-recognition molecules initiating the lectin pathway of complement is ficolin-2. In a large cohort of neonates (recruited consecutively, including approx. 18% preterms), relative ficolin-2 insufficiency was shown to be associated with prematurity, low birthweight and perinatal infections (15). Ficolin-2 was also demonstrated to contribute to the clearance of serotype III group B streptococci – a common cause of neonatal sepsis (16), often clinically and radiographically indistinguishable from RDS (17). In women diagnosed with pre-eclampsia, serum ficolin-2 concentration was significantly lower in comparison with uncomplicated pregnancies (18, 19). Moreover, deposition of ficolin-2 on apoptotic trophoblasts in preeclampsia was observed (18).

So far, published reports have not demonstrated a strong relationship between prematurity-associated complications and the frequency of promoter or exon 8 *FCN2* gene variants, known to markedly influence ficolin-2 level/activity (20). Recently we analysed 15 polymorphisms localised to the *FCN2* 3’untranslated region (3’UTR) in a large group of preterm babies. Our data revealed that reconstructed diplotypes, including both the G variant at rs4521835 and the C variant at rs73664188 (classified into proposed group VI) were associated with a significantly lower ficolin-2 concentration in cord serum (21).

Ficolin-2 is considered to be an important factor of innate immunity. Several reports suggested its association not only with infections but also with non-infectious diseases and its potential application as a prognostic marker (22–24). In this study, we examined ficolin-2 levels in cord-blood serum of preterm neonates in the context of prematurity-associated complications and its possible usefulness for their diagnosis. Moreover, the genetically determined origin of the differences in ficolin-2 levels observed was verified *via* analysis of *FCN2* gene polymorphisms.

## 2 Material and methods

### 2.1 Subjects

Cord blood samples from 546 Polish preterm neonates, including 118 born at gestational age (GA) <33^rd^ weeks (range 24-32 weeks), and 428 born between 33^rd^ and 37^th^ weeks of pregnancy were obtained from the Department of Newborns’ Infectious Diseases (Poznań University of Medical Sciences, Poland), Department of Neonatology (Medical University of Gdańsk, Poland) and Department of Perinatology (Medical University of Łódź, Poland). This cohort included 504 subjects investigated previously (21).

The basic clinical data are presented in **Table 1**. The clinical data for neonates separated into the subgroups born at GA <33 and GA ≥33 are presented in **Supplementary Table 1**. The study was approved by the corresponding local ethics committees (Bioethics Committee of The Karol Marcinkowski Poznań University of Medical Sciences, Independent Bioethics Committee for Scientific Research at Medical University of Gdańsk, Bioethics Committee of The Medical University of Łódź). Written informed parental consent was obtained. This work conforms to the provisions of the Declaration of Helsinki.

**Table 1:** Clinical data of preterm neonates included into the study (n=546).

Variable	N (%)
Sex	M: 285 (52.2) F: 261 (47.8)
GA <33 GA ≥33	118 (21.6%) 428 (78.4%)
Birthweight <1500 g	62 (11.4)
Multiple pregnancy	106 (19.4)
Gestational diabetes mellitus (GDM)	90 (16.5)
Hypertension in mother	70 (12.8)
Pre-eclampsia	25 (4.6)
Preterm premature rupture of membranes (pPROM)^1^	99 (18.1)
Antenatal corticosteroid therapy^2^	221 (40.4)
Caesarean section	349 (63.9)
Early-onset infection (EOI)^3^	81 (14.8)
Respiratory distress syndrome (RDS)^4^	140 (25.6)
Patent ductus arteriosus (PDA)	14 (2.6)
Tachycardia^5^	36 (6.6)
Necrotizing enterocolitis (NEC)	17 (3.1)
Perinatal hypoxia^6^	20 (3.7)
Respiratory support^7^	163 (29.9)
Delivery room intubation	31 (5.7)
Mean length of hospitalisation (days)^8^	15.3
Mean length of stay at NICU (days)^8^	5.0

^1-^more than 24 h before delivery, ^2^- at least one course, ^3^- caused by variety of agents (including *staphylococci, E. coli, Klebsiella* sp. and GBS; in majority of cases however, agents were not identified and infection was diagnosed basing on clinical symptoms), ^4^- respiratory distress with an oxygen requirement to maintain oxygen saturations of ≥90%, accompanied by a characteristic chest radiograph, ^5^- >160 per min, ^6^- pH ≤7.0 or BE ≤12 mM/ml in umbilical artery or within 1h of life, ^7^- mechanical ventilation, continuous positive airway pressure (CPAP) or both, ^8^- after excluding three cases of death

Cord samples were taken at birth into sodium citrate (for DNA isolation) and clot activator (for serum) containing tubes. Isolated serum was kept at -80°C. DNA was isolated using GeneMATRIX Quick Blood Purification Kit (EURx Ltd. Gdańsk, Poland), according to the manufacturer’s protocol.

### 2.2 Determination of ficolin-2 concentration in cord sera

Ficolin-2 concentrations in 481 cord blood serum samples were determined by TRIFMA, as described by Świerzko et al. (25). Briefly, anti-ficolin-2 mAb (ABS 005-16, BioPorto Diagnostics, Denmark) for 384 HB Optiplate coating, whereas biotinylated mAb (GN4, Hycult Biotech, The Netherlands) and Eu^3+^-labelled streptavidin (Perkin Elmer, USA) for protein detection were used. The 25^th^ percentile (determined for the whole group or for subgroups of neonates born at GA <33 and at GA ≥33) was taken as an indicator of low ficolin-2 concentration. Although the influence of tubes used for blood collection on concentration of ficolin-2 determined in serum was evidenced (26, 27), tubes of the same type were used in all three clinics collecting samples what makes all data fully comparable.

### 2.3 Determination of *FCN2* polymorphisms

The differences in ficolin-2 levels were analysed in the context of the *FCN2* gene promoter haplotype GGCA [corresponding to single nucleotide polymorphisms at positions: -986 (rs3124952); -602 (rs3124953); -64 (rs7865453); -4 (rs17514136)] (28) and 3’UTR group VI diplotype (21), both associated with low ficolin-2 concentration.

Briefly, promoter polymorphisms at positions -986 and -602 were investigated by PCR-RFLP analysis in 503 DNA samples, according to the procedures published by Metzger et al. (29). SNP at positions -64 and -4 were determined using allele-specific PCR or PCR-RFLP, respectively, as described by Szala et al. (30), with minor modifications. Polymorphisms in the *FCN2* 3’UTR were determined *via* Sanger sequencing or allelic discrimination using TaqMan probes (21). Haplotypes and diplotypes were created using Haploview 4.2 and PHASE software.

### 2.4 Statistical analysis

To determine whether continuous variables had a normal distribution, the Shapiro–Wilk test was conducted. The ficolin-2 concentrations were compared using Mann-Whitney *U* test or Kruskal-Wallis test with *post-hoc* Dunn test, depending on the number of compared groups. Spearman’s rank correlation test assessed the correlations. The frequencies of genotypes/clinical complications were compared by Fischer’s exact or χ^2^ test when appropriate. Odds ratios were calculated using online MedCalc software (https://www.medcalc.org). Clinical associations of low ficolin-2 concentration were verified using multiple logistic regression analysis. The predictive power of ficolin-2 was evaluated by receiver operating characteristics (ROC) curves and area under the curve (AUC) analysis. The Statistica (version 13.3, TIBCO Software) and SigmaPlot (version 12, Systat Software) software were used for data management and statistical calculations. P values <0.05 were considered statistically significant.

## 3 Results

### 3.1 Low ficolin-2 levels in the context of basic clinical characteristics and outcomes

Median ficolin-2 concentrations differed markedly between neonates born before the 33^rd^ week of gestation and those born at GA ≥33 (median 1639 ng/ml *vs* 2036 ng/ml, p=0.0024) (**Supplementary Figure 1A**). Moreover, in neonates with very low birthweight (VLBW, <1500 g), ficolin-2 was significantly lower than in babies with higher birthweight (median 1496 ng/ml *vs* 2018 ng/ml, p=0.0001) (**Supplementary Figure 1B**).

Weak but significant correlations of ficolin-2 concentration with Apgar 1’ and Apgar 5’ scores were observed (R=0.18, p=0.0002 and R=0.24, p<0.0001, respectively). The median ficolin-2 level for babies with Apgar 1’ score <7 (1559 ng/ml) was lower than that for those with Apgar 1’ score ≥7 (2014 ng/ml, p=0.01) (**Supplementary Figure 1C**). In the case of Apgar 5’, medians were 1409 ng/ml and 2042 ng/ml, respectively (p=0.0098) (**Supplementary Figure 1D**).

Moreover, ficolin-2 inversely correlated with the length of hospitalisation (R=-0,2, p<0,0001) and the length of stay at the neonatal intensive care unit (NICU) (R=-0.13, p=0.007). In neonates who stayed in the hospital for >2 weeks, the median ficolin-2 concentration was significantly lower (1646 ng/ml) than in babies, who stayed in the hospital for a shorter time (2094 ng/ml, p=0.0001) (**Supplementary Figure 1E**). In the group of neonates with ficolin-2 levels below 1295 ng/ml (<25^th^ percentile), the length of stay at the neonatal ward was generally longer (mean 18 days, range 2-78 days) than in those with higher ficolin-2 (mean 14 days, range 1-91 days, p=0.02). Furthermore, ficolin-2 concentration was significantly lower (1749 ng/ml) in neonates who stayed at least 5 days at NICU in comparison with babies who stayed there for up to 4 days or did not need intensive care (2018 ng/ml, p=0.022) (**Supplementary Figure 1F**). It is worth noting that all neonates with ficolin-2 concentration <25^th^ percentile and born at GA <33 required hospitalisation at NICU and their stay was generally longer than that of newborns with the same GA but with higher ficolin-2 levels [22.8 days (range 4-61 days) *vs.* 14.9 days (range 0-84 days), p=0.002).

### 3.2 Low ficolin-2 levels in the context of complications associated with prematurity

The data concerning relationships between low (<25^th^ percentile) ficolin-2 concentration in the cord blood and selected prenatal clinical complications of prematurity in the whole cohort are presented in **Table 2**. Additionally, *FCN2* gene promoter haplotype GGCA and 3’UTR diplotype group VI were included in the analysis. Corresponding analyses between subgroups depending on gestational age (<33 weeks and ≥33 weeks, in the context of a wider panel of complications) are presented in **Supplementary Table 2**.

**Table 2:** Univariate and multivariate^1^ regression analysis of potential factors for low ficolin-2 concentration (below 25^th^ percentile)^2^ in the whole cohort.

Variable	Univariate analysis			Multivariate analysis		
	OR	95%CI	*p*	OR	95%CI	*p*
GA <33 weeks	1.33	0.82-2.15	0.2478	**-**	**-**	**-**
Birthweight <1500 g	**2.12**	**1.17-3.84**	**0.0126**	**2.14**	**1.14-4.02**	**0.0177**
Promoter GGCA haplotype	**3.63**	**2.06-6.40**	**<0.0001**	**2.57**	**1.26-5.26**	**0.0098**
3’UTR diplotype group VI	**2.46**	**1.54-3.93**	**0.0002**	1.51	0.85-2.70	0.1641
Sex (Male)	1.31	0.86-2.01	0.2107	–	–	–
GDM	1.01	0.58-1.79	0.9634	–	–	–
pPROM	**0.55**	**0.31-0.99**	**0.0460**	0.55	0.29-1.05	0.0685
Fetal growth restriction (FGR)	1.55	0.83-2.88	0.1698	–	–	–
Intrauterine hypoxia	0.81	0.22-2.96	0.7512	–	–	–
Hypertension in pregnancy	1.17	0.63-2.17	0.6134	–	–	–

^1^- multivariate analysis data are shown when p<0.1 in univariate analysis; ^1^- 1295 ng/ml. The values are marked in bold when statistically significant.

Logistic univariate regression analysis revealed associations of low ficolin-2 with VLBW, *FCN2* promoter GGCA haplotype and group VI 3’UTR diplotype (**Table 2**). In contrast, premature rupture of membranes (pPROM) was associated with higher ficolin-2 concentrations. Multivariate analysis confirmed that low birthweight [OR=2.14, 95%CI (1.14-4.02), p=0.018] and GGCA haplotype [OR=2.57, 95%CI (1.26-5.26), p=0.0098], (but not the group VI 3’UTR diplotype) independently predicted low ficolin-2 concentration (**Table 2**).

### 3.3 Low ficolin-2 levels in the context of respiratory distress syndrome

Next, the univariate and multivariate logistic regression analysis for RDS as the dependent variable was performed (**Table 3**). According to the final model, the likelihood of RDS development was increased in neonates with low ficolin-2 cord blood serum concentration [OR=2.05, 95% CI (1.24-3.37), p=0.0051]. It was also associated with shorter GA [OR=7.64, 95% CI: (4.34-13.43), p<0.0001], very low birthweight [OR=3.2, 95% CI (1.46-7.03), p=0.0037], and PDA [OR=11.04, 95% CI (1.87-65.02), p=0.008].

**Table 3:** Univariate and multivariate logistic regression analysis^1^ of potential factors for RDS development.

Variable	Univariate analysis			Multivariate analysis		
	OR	95%CI	*p*	OR	95%CI	*p*
**GA <33 weeks**	**11.41**	**7.14-18.23**	**<0.0001**	**7.64**	**4.34-13.43**	**<0.0001**
**Birthweight <1500 g**	**9.75**	**5.39-17.63**	**<0.0001**	**3.20**	**1.46-7.03**	**0.0037**
**Low ficolin-2**	**2.51**	**1.64-3.85**	**<0.0001**	**2.05**	**1.24-3.37**	**0.0051**
Promoter GGCA haplotype	1.33	0.75-2.35	0.326	–	–	–
3’UTR diplotype group VI	1.31	0.82-2.07	0.255	–	–	–
Sex (Male)	1.24	0.84-1.84	0.273	–	–	–
Caesarean section	1.45	0.95-2.20	0.081	1.36	0.81-2.30	0.247
GDM	1.37	0.84-2.25	0.207	–	–	–
Preeclampsia	1.66	0.72-3.85	0.236	–	–	–
pPROM	1.11	0.68-1.82	0.667	–	–	–
Fetal growth restriction (FGR)	0.98	0.54-1.80	0.956	–	–	–
**NEC**	**13.65**	**3.83-48.66**	**0.0001**	3.90	0.86-17.57	0.0767
**PDA**	**18.80**	**4.15-85.1**	**0.0001**	**11.04**	**1.87-65.02**	**0.008**
Tachycardia	1.23	0.57-2.65	0.59	–	–	–
Hypertension in pregnancy	1.01	0.57-1.80	0.966	–	–	–

^1^- multivariate analysis data are shown when p<0.1 in univariate analysis. The values are marked in bold when statistically significant.

**Figure 1:**
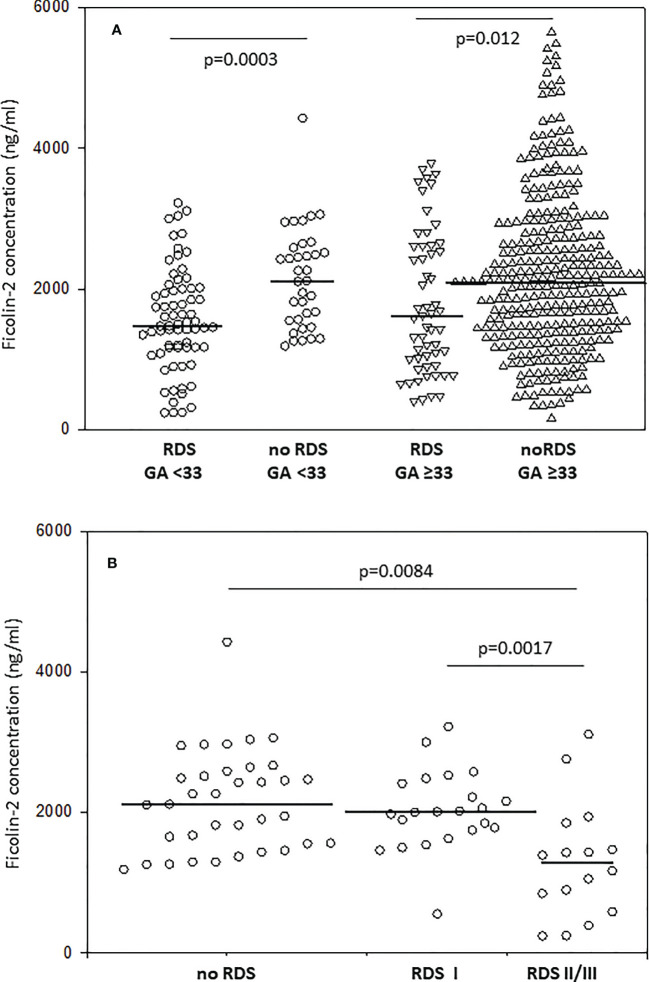
The comparison of ficolin-2 cord blood serum concentration in preterm neonates with and without respiratory distress syndrome (RDS), depending on gestational age **(A)** and disease severity in neonates delivered at GA <33 **(B)**. The horizontal lines mark median values. Medians were compared with the use of Mann-Whitney *U* test **(A)** and Kruskal-Wallis test (p=0.015) with *post-hoc* Dunn test **(B)**.

Median ficolin-2 concentration was significantly lower in newborns diagnosed with RDS compared with neonates without this complication (for patients born <33 GA: 1471 *vs.* 2115 ng/ml, p=0.0003; for patients born ≥33 GA: 1610 *vs*. 2081 ng/ml; p=0.012) (**Figure 1A**). For thirty-eight neonates with RDS born at GA <33, data about disease severity were available. Kruskal-Wallis test revealed a significant difference in ficolin-2 levels among groups of babies with no RDS, those with mild and more advanced RDS (p=0.015) (medians: 2115 ng/ml, 2002 ng/ml and 1275 ng/ml, respectively). The *post-hoc* analysis showed significantly lower ficolin-2 in cord blood serum in neonates diagnosed with more advanced compared with those with mild RDS (p=0.0017) or without this complication (p=0.0084) (**Figure 1B**).

**Figure 2:**
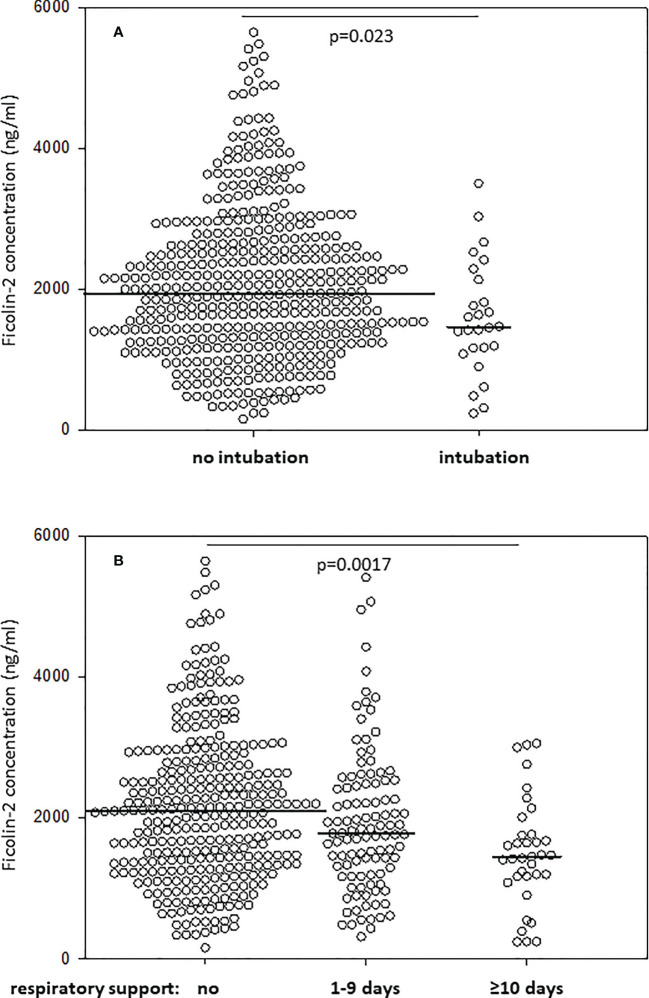
Comparison of ficolin-2 concentration in neonates requiring and not requiring intubation at delivery room **(A)** and/or respiratory support (mechanical ventilation, CPAP, or both) **(B)**. The horizontal lines mark median values. Medians were compared with the use of Mann-Whitney *U* test **(A)** and Kruskal-Wallis test (p=0.001) with *post-hoc* Dunn test **(B)**.

The management of RDS includes exogenous surfactant therapy (by intubation in the delivery room) as well as assisted ventilation. Ficolin-2 concentrations were lower in babies requiring intubation in the delivery room than in those who did not require it (1461 *vs.* 1938 ng/ml, p=0.023) (**Figure 2A**). The duration of respiratory support (mechanical ventilation and/or continuous positive airway pressure, CPAP) inversely correlated with ficolin-2 concentration in cord serum (R=-0.154, p<0.001).

Furthermore, Kruskal-Wallis analysis showed a statistically significant difference in the level of ficolin-2 across three groups: without respiratory support, with respiratory support duration <10 days and with respiratory support ≥10 days (p=0.001) (**Figure 2B**). Patients who did not require respiratory support had higher median ficolin-2 concentration (median 2089 ng/ml) compared with patients requiring ≥10 days of respiratory support (median 1447 ng/ml, *post-hoc* Dunn test p=0.0017) but compared with neonates on <10 days of respiratory support the difference was not statistically significant (median 1782 ng/ml, p=0.208).

**Figure 3:**
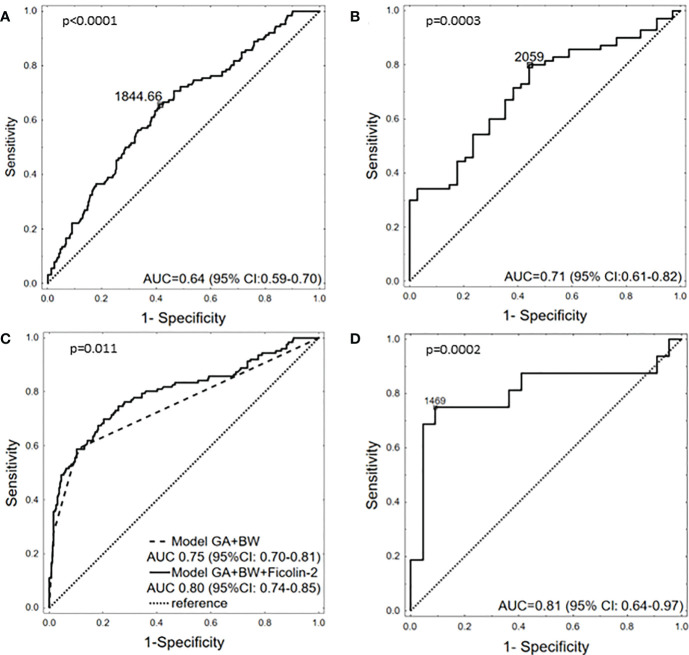
The potency of ficolin-2 to differentiate between babies with and without RDS in whole cohort **(A)**, between neonates with and without RDS, born at GA <33 **(B)**, between neonates with and without RDS, depending on GA (<33 *vs.* ≥33) and BW (<1500g *vs.* ≥1500g) **(C)** and between babies born at GA <33 with mild **(I)** and moderate/severe (II+III) RDS **(D)**.

The potential of ficolin-2 to discriminate between patients with and without RDS was also tested. When all samples were analysed, the selective power of ficolin-2 appeared rather low [AUC=0.643, 95% CI (0.588-0.699), p<0.0001] (**Figure 3A**). When analysis was performed for neonates born at GA <33 only, it increased to AUC=0.712 [95% CI (0.612-0.817), p=0.0003] (**Figure 3B**). Then, we developed a simple logistic regression model with GA and birthweight (≥/<1500 g) as independent variables. This model yielded AUC of 0.75 [95% CI (0.70-0.81, p<0.001] with a sensitivity 59.5% and specificity 88.0%. Ficolin-2 level incorporation into the model significantly increased its performance up to AUC 0.80 [95% CI (0.74-0.85), p=0.011) (**Figure 3C**). Importantly, sensitivity of this model reached 69.1% with preserved specificity (79.8%). Furthermore, ficolin-2 concentration could better differentiate between mild and moderate/severe RDS in newborns born at GA <33 [AUC=0.807, 95% CI (0.644-0.97), p=0.0002] with sensitivity of 75% and specificity of 91% at cut off 1469 ng/ml (**Figure 3D**). It should however be remembered that the number of samples with corresponding data concerning disease severity was rather small.

## 4 Discussion

The main goal of this work was to investigate the association of ficolin-2 concentration at birth with the risk of complications related to prematurity. We demonstrated that low (<25^th^ percentile) ficolin-2 is associated with a lower Apgar score, prolonged hospitalisation and stay at NICU. Moreover, a strong relationship with increased probability of RDS was found (**Table 3**). Therefore, immediately after birth preterms with low ficolin-2 levels are generally in poorer condition than those with higher concentrations of this protein.

RDS was originally described as hyaline membrane disease- (HMD)-associated vascular disruption leading to the leakage of plasma into the alveolar spaces and layering of fibrin and necrotic cells arising from type II pneumocytes along the alveolar ductus (6). It develops at or within 24 h after birth, and causes hypoxia in association with a lack of surfactant. Untreated disease leads to severe hypoxia resulting in multiple organ failure and even death.

Lung injury and progression to pulmonary edema are also suspected to be consequences of the activation of complement and its cross-talk with the coagulation system (31, 32). Several reports have described lower concentrations of complement factors and higher levels of complement activation products in neonates with RDS, especially in the subgroup poorly responding to surfactant treatment compared with good responders (33). Elevated C3a was also proposed to differentiate RDS from RDS accompanied by perinatal asphyxia (34). Knowledge concerning the role of lectin pathway factors in RDS is limited to a single report by Dogan et al. (35), showing significantly higher frequency of *MBL2* genotypes associated with low concentration of MBL in affected neonates. Our results showing increased probability of development of RDS in preterms with low ficolin-2 confirm an association of the complement system with this complication. Interestingly, we found no significant relationship between *FCN2* gene polymorphisms and RDS.

Respiratory distress syndrome may be prevented by corticosteroid administration and its treatment usually requires mechanical or non-invasive ventilation and transbronchial exogenous surfactant application. However, it may still result in the development of neonatal chronic lung disease and possibly severe long-term lung damage. Selection of specific and sensitive markers of RDS could be helpful in the diagnostic process and in choosing appropriate treatment. Despite intensive studies, so far no such marker is available. Our results may be helpful in this context since they suggest that ficolin-2 insufficiency has the potential to differentiate between preterms with and without RDS as well as those with mild or more severe disease, at least in the subgroup born at GA<33 (**Figure 3**). Moreover, we reported that low ficolin-2 in cord blood may predict a need for intubation in the delivery room as well as a requirement for intensive respiratory support.

Several reports evidenced association of low ficolin-2 concentration with diseases of the respiratory system. For example, relative ficolin-2 deficiency was shown to predispose to the development of bronchiectasis (36) and to predict disease progression in patients with idiopathic pulmonary fibrosis (37). Although, no association of ficolin-2 with susceptibility to community-acquired pneumonia was shown (38), its low concentration may increase the risk of infection with *Mycobacterium tuberculosis* or *M. avium* complex (39, 40). Median ficolin-2 level was also demonstrated to be significantly lower in paediatric patients with asthma and/or allergic rhinitis suffering from recurrent respiratory infections than in controls (41). Interestingly, Schaubel et al. (42) reported that neonatal RDS (with and without BPD) significantly enhances the risk of asthma in pre-school-aged children. Although ficolin-2 is synthesised by hepatocytes and secreted into the circulation, it can be present in the lung as well (43, 44). Interestingly, very low *FCN2* mRNA was found in the fetal lung (45).

Low circulating ficolin-2 in RDS patients may be inborn or be a consequence of its consumption or leakage into the air space due to impaired function of the alveolar-capillary barrier. It may lead not only to complement activation, but also to activation of the coagulation system, *via* complexed MASP (MBL-associated serine proteases). Purified ficolin-2-MASP complexes were shown to release fibrinopeptides from fibrinogen, activate factor XIII and in consequence, to form a clot (46). It is also possible that ficolin-2 interacts with lung surfactant components like cholesterol crystals which constitute up to 8% of native lung surfactant. Ficolin-2-MASP complex deposition, followed by complement C4 activation on cholesterol crystals was documented by Pilely et al. (47). On the other hand, cholesterol is a major component of meconium - involved also in respiratory distress pathology and mentioned among factors responsible for surfactant inactivation (48).

Apoptosis was proposed to contribute to the pathogenesis of RDS, since numerous apoptotic cells were detected mainly in the respiratory epithelium in lungs of affected infants (49). Late apoptotic/necrotic cells may be also a target for ficolin-2/ficolin-2-MASP complexes resulting in complement activation and enhanced phagocytosis (50, 51).

Our data confirmed association of the *FCN2* gene promoter polymorphisms and ficolin-2 concentration, published previously by others and ourselves (20, 28, 52). We previously reported also the relationship of 3’UTR diplotypes (group VI) with low ficolin-2 (21). That was confirmed here in univariate analysis, however lost statistical significance after multivariate logistic regression. It has to be remembered as well that prenatal steroid administration might affect ficolin-2 concentration in cord serum. We found a difference between babies born at GA ≥33 to mothers treated with steroids *vs.* those of mothers who were not receiving such a treatment, which however was not confirmed in multivariate regression analysis (**Supplementary Table 2B**).

We report here for the first time possible involvement of ficolin-2 insufficiency in neonatal RDS development. However, it is not clear whether low concentration in cord blood is a cause or consequence of disease progression. It can be assumed that cord blood ficolin-2 <1500 ng/ml may enhance the probability of moderate/severe RDS and the need for surfactant therapy and assisted ventilation. Determination of ficolin-2 concentration in cord serum may be considered a new early prognostic factor in RDS development, helpful to distinguish RDS from other prematurity-associated respiratory disorders and thus facilitating the choice of appropriate treatment. That, however, should be confirmed by an independent study, ideally taking into account responsiveness to treatment and changes of ficolin-2 levels during the course of RDS.

## Data availability statement

The raw data supporting the conclusions of this article will be made available by the authors, without undue reservation.

## Ethics statement

The studies involving human participants were reviewed and approved by Bioethics Committee of The Karol Marcinkowski Poznań University of Medical Sciences, Independent Bioethics Committee for Scientific Research at Medical University of Gdańsk, Bioethics Committee of The Medical University of Łódź. Written informed consent to participate in this study was provided by the participants’ legal guardian/next of kin.

## Author contributions

GG and AŚ determined ficolin-2 concentrations; AS-P, GG, AŚ, and DJ determined *FCN2* gene promoter polymorphisms. AŚ coordinated project realisation, designed the study, planned and supervised experimental work; DJ sequenced *FCN2* gene 3’UTR; PK, KC, MK-B, and KS were responsible for patients’ qualification, taking and collecting samples as well as collecting clinical data; ID-P, JM, and JK supervised qualification and recruitment of patients and analysis of clinical data; MM and HS provided anti-ficolin-2 mAbs for initial experiments, contributed to modification of the procedure and discussed corresponding data; DM, JC, WF, and MC performed statistical analysis; MC contributed to the study design, supervision of experimental work and analysed promoter haplotypes. The draft manuscript was written by AŚ and MC, discussed, corrected and finally approved by all authors. All authors contributed to the article and approved the submitted version.
