# Supplementary material for: Association of low ficolin-2 concentration in cord serum with respiratory distress syndrome in preterm newborns
Source: Front Immunol. 2023 Jan 17;14:1107063. doi: 10.3389/fimmu.2023.1107063 (PMC9886859; doi:10.3389/fimmu.2023.1107063)
Supplement: Supplementary file 1 [file DataSheet_1.pdf]

Supplementary Material

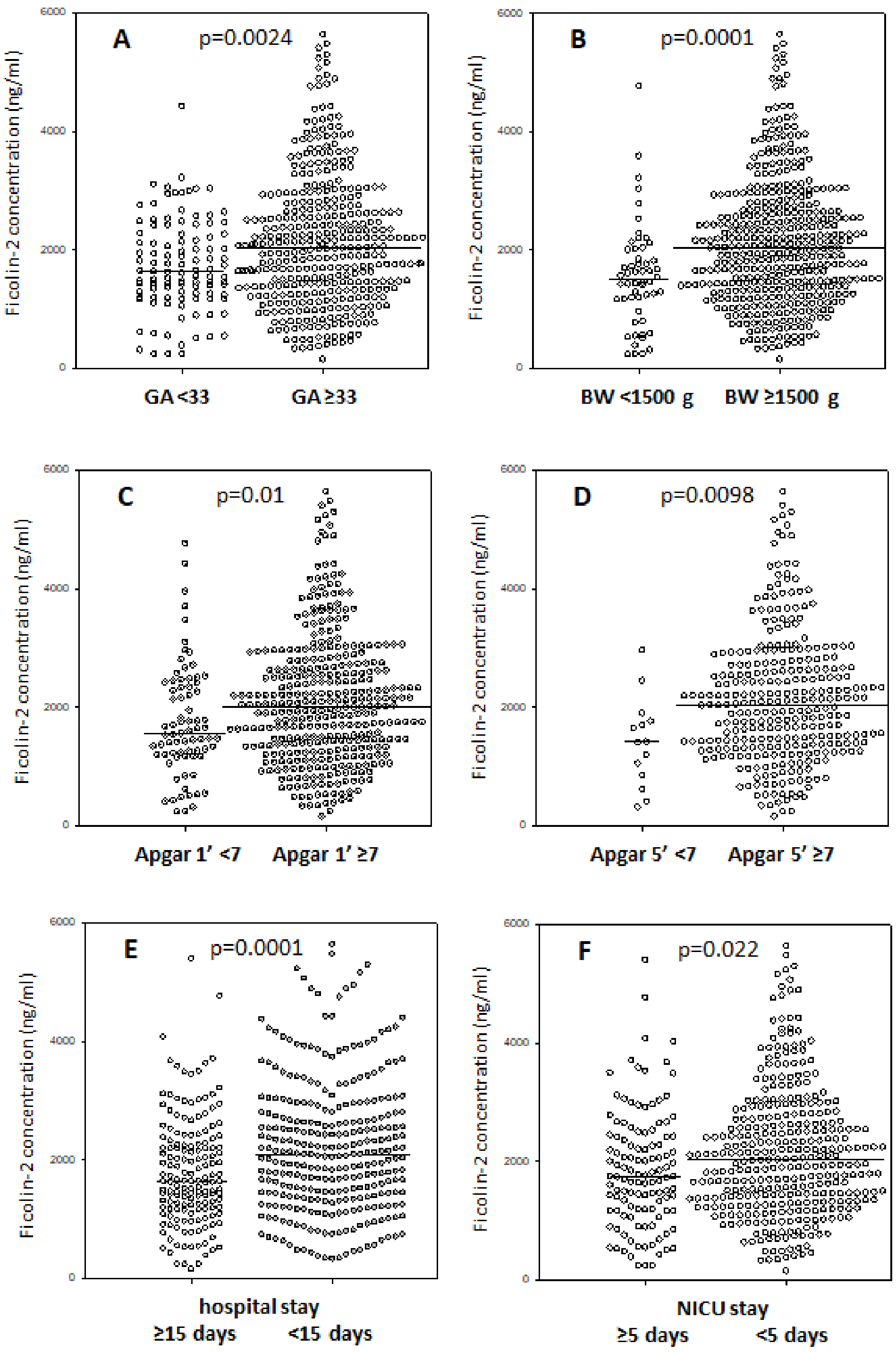

Supplementary Figure 1. Comparison of ficolin-2 concentrations in preterm neonates depending on clinical parameters: **A** - gestational age; **B** - birthweight; **C**- 1' Apgar score; **D** - 5' Apgar score; **E** – duration of hospital stay; **F** - duration of stay at NICU. Black bars indicate median values.

Supplementary Table 1 Clinical data of preterm neonates included into the study, depending on gestational age.

Variable	Gestational age <33 weeks	Gestational age ≥33 weeks	*p*-value
N (%)	118 (100)	428 (100)	-
Sex (Male)	57 (48.3)	228 (53.3)	0.723
Birthweight <1500g	46 (39.0)	16 (3.7)	p<0.0001
Multiple pregnancy	24 (20.3)	82 (19.2)	0.797
Gestational diabetes mellitus (GDM)	18 (15.3)	72 (16.8)	0.1
Hypertension in mother	9 (7.6)	61 (14.3)	0.08
Pre-eclampsia	3 (2.5)	22 (5.1)	0.325
Preterm premature rupture of membranes (pPROM)[1]	25 (21.2)	74 (17.3)	1
Antenatal corticosteroid therapy[2]	100 (84.7)	118 (27.6)	p<0.0001
Caesarean section	77 (65.2)	272 (63.6)	0.66
Early-onset infection (EOI)	42 (35.6)	39 (9.1)	p<0.0001
Respiratory distress syndrome (RDS)[3]	77 (65.2)	63 (14.7)	p<0.0001
Patent ductus arteriosus (PDA)	8 (6.8)	6 (1.4)	0.003
Tachycardia[4]	19 (16.1)	16 (3.7)	<0.0001
Necrotizing enterocolitis (NEC)	14 (11.9)	3 (0.7)	<0.0001
Perinatal hypoxia[5]	2 (1.7)	16 (3.7)	0.39
Respiratory support[6]	95 (82)	71 (16.6)	<0.0001
Delivery room intubation	25 (21.1)	6 (1.4)	<0.0001
Days to hospital discharge[7] Mean (range)	33 (3-91)	10.5 (1-64)	<0.0001
Days at ICU[7] Mean (range)	16.5 (0-84)	1.9 (0-56)	<0.0001

[1]- more than 24 h before delivery, [2]- at least one course, [3]- respiratory distress with an oxygen requirement to maintain oxygen saturations of ≥90%, accompanied by a characteristic chest radiograph, [4]- >160 per min, [5]- pH ≤7.0 or BE ≤12 mMol/ml in umbilical artery or within 1h of life, [6]- mechanical ventilation, CPAP or both, [7]- after excluding three cases of death

Supplementary Table 2. Univariate and multivariate regression analysis for potential predictors for low ficolin-2 concentration (below 25th percentile) in the group of neonates born preterm [<33 weeks of gestation (**A**) and ≥ 33 weeks of gestation (**B**)].

A

Variable	<33 GA[1]						
	N	Univariate analysis			Multivariate analysis[2]		
		OR	95% CI	*p*-value	OR	95% CI	*p*-value
BW <1500 g	46	2.01	0.81-4.99	0.132	-	-	-
Promoter GGCA haplotype	16	2.51	0.78-8.1	0.123	-	-	-
3'UTR diplotype group VI	25	**3.51**	**1.3-9.46**	**0.013**	2.38	0.61-9.3	0.219
Sex (Male)	57	1.44	0.57-3.62	0.442	-	-	-
Caesarean section	77	1.3	0.5-3.38	0.589	-	-	
GDM	18	1.6	0.49-5.05	0.45	-	-	-
Pre-eclampsia	3[4]	-	-	-	-	-	-
Hypertension in pregnancy	9	3.62	0.83-15.7	0.086	-	-	-
pPROM	25	0.54	0.17-1.74	0.299	-	-	-
Fetal growth restriction (FGR)	11	0.27	0.07-1.02	0.054	-	-	-
Tachycardia	19	0.36	0.08-1.71	0.2	-	-	-
Perinatal hypoxia	2	3.29	0.2-54.63	0.406	-	-	-
Prenatal steroid therapy	100	2.03	0.42-9.75	0.377	-	-	-
NEC	14	0.86	0.22-3.35	0.822	-	-	-
PDA	8	0.79	0.08-7.43	0.838	-	-	-
**RDS**	**77**	**16.15**	**2.08-125.57**	**0.0079**	**17.51**	**2.23-137.68**	**0.0065**
EOI	42	1.54	0.62-3.85	0.356	-	-	-
Pneumonia	35	2.07	0.82-5.25	0.125	-	-	-
SIRS	2[3]	-	-	-	-	-	-

[1]- ficolin-2 concentration <1237 ng/ml; [2]- multivariate analysis data are shown when p<0.05 in univariate analysis; [3]-not analysed

B

**Variable**	**≥ 33 GA**[1]						
	N	Univariate analysis			Multivariate analysis[2]		
		OR	95% CI	*p*-value	OR	95% CI	*p*-value
BW <1500 g	16	1.36	0.41-4.52	0.62	-	-	-
Promoter GGCA haplotype	**49**	**3.33**	**1.74-6.37**	**0.0003**	2.31	1-5.375	0.051
3'UTR diplotype group VI	94	**2.64**	**1.55-4.49**	**0.0004**	1.66	0.84-3.28	0.143
Sex (Male)	228	1.29	0.8-2.09	0.301	-	-	-
Caesarean section	272	1.11	0.68-1.8	0.687	-	-	-
GDM	72	1.12	0.6-2.11	0.715	-	-	-
Pre-eclampsia	22	0.52	0.15-1.81	0.304	-	-	-
Hypertension in pregnancy	61	0.95	0.47-1.9	0.879	-	-	-
**pPROM**	**74**	**0.44**	**0.22-0.91**	**0.026**	**0.31**	**0.17-0.88**	**0.023**
Fetal growth restriction (FGR)	51	1.24	0.61-2.54	0.555	-	-	-
Tachycardia	16	0.6	0.13-2.77	0.509	-	-	-
Perinatal hypoxia	16	0.49	0.11-2.24	0.358	-	-	-
Prenatal steroid therapy	**118**	**1.82**	**1.1-2.98**	**0.018**	1.44	0.822.55	0.205
NEC	3[3]	-	-	-	-	-	-
PDA	6	1.52	0.27-8.45	0.63	-	-	-
**RDS**	**63**	**2.47**	**1.36-4.48**	**0.0029**	**2.52**	**1.3-4.88**	**0.006**
EOI	39	1.13	0.52-2.43	0.76	-	-	-
Pneumonia	15	0.82	0.22-3	0.76	-	-	-
SIRS	4	9.44	0.97-91.9	0.053	-	-	-

[1]- ficolin-2 concentration <1321 ng/ml; [2] - multivariate analysis data are shown when p<0.05 in univariate analysis; [3]-not analysed
